# Biochemical and structural studies reveal differences and commonalities among cap-snatching endonucleases from segmented negative-strand RNA viruses

**DOI:** 10.1074/jbc.RA118.004373

**Published:** 2018-10-22

**Authors:** Tobias Holm, Janine-Denise Kopicki, Carola Busch, Silke Olschewski, Maria Rosenthal, Charlotte Uetrecht, Stephan Günther, Sophia Reindl

**Affiliations:** From the ‡ Department of Virology, Bernhard-Nocht-Institute for Tropical Medicine, 20359 Hamburg, Germany,; the § Heinrich Pette Institute, Leibniz Institute for Experimental Virology, 20251 Hamburg, Germany, and; the ¶ European XFEL GmbH, 22869 Schenefeld, Germany

**Keywords:** negative-strand RNA virus, viral transcription, endonuclease, enzyme structure, metal ion–protein interaction, recombinant protein expression, small-angle X-ray scattering (SAXS), structure-function, cap-snatching, nairovirus

## Abstract

Viruses rely on many host cell processes, including the cellular transcription machinery. Segmented negative-strand RNA viruses (sNSV) in particular cannot synthesize the 5′-cap structure for their mRNA but cleave off cellular caps and use the resulting oligonucleotides as primers for their transcription. This cap-snatching mechanism, involving a viral cap-binding site and RNA endonuclease, is both virus-specific and essential for viral proliferation and therefore represents an attractive drug target. Here, we present biochemical and structural results on the putative cap-snatching endonuclease of Crimean–Congo hemorrhagic fever virus (CCHFV), a highly pathogenic bunyavirus belonging to the Nairoviridae family, and of two additional nairoviruses, Erve virus (EREV) and Nairobi sheep disease virus (NSDV). Our findings are presented in the context of other cap-snatching endonucleases, such as the enzymatically active endonuclease from Rift Valley fever virus (RVFV), from Arenaviridae and Bunyavirales, belonging to the His− and His+ endonucleases, respectively, according to the absence or presence of a metal ion–coordinating histidine in the active site. Mutational and metal-binding experiments revealed the presence of only acidic metal-coordinating residues in the active site of the CCHFV domain and a unique active-site conformation that was intermediate between those of His+ and His− endonucleases. On the basis of small-angle X-ray scattering (SAXS) and homology modeling results, we propose a protein topology for the CCHFV domain that, despite its larger size, has a structure overall similar to those of related endonucleases. These results suggest structural and functional conservation of the cap-snatching mechanism among sNSVs.

## Introduction

Crimean–Congo hemorrhagic fever virus (CCHFV)^3^ is a tick-borne virus within the Nairoviridae family of the order Bunyavirales. It is distributed over a large geographical region ranging from South Africa to southeastern Europe and from West Africa to China (1). It infects livestock and humans and can cause severe hemorrhagic fever with a mortality rate of 30% (2). The virus is transmitted to humans by either tick bites or direct contact with contaminated blood or tissue samples from the infected hosts. All members of the order Bunyavirales possess three negative sense RNA genome segments named S, M, and L segments. They encode the nucleocapsid protein (N protein), the glycoprotein precursor, and the large L protein, which contains the viral RNA-dependent RNA polymerase (RdRp), respectively (3, 4). Like all segmented negative strand viruses (sNSVs), nairoviruses carry out transcription using the cap-snatching mechanism, which requires the presence of a metal ion–dependent RNA endonuclease (5, 6). This endonuclease was identified at the very N terminus of many different L proteins, *e.g.* Lassa virus (7), lymphocytic choriomeningitis virus (LCMV) (8) (Arenaviridae), Andes virus (ANDV) (9), Hantaan virus (HNTV) (10) (Hantaviridae), and La Crosse virus (LACV) (11) (Peribunyaviridae) and at the very N terminus of the PA subunit of the influenza virus polymerase complex (12) (Orthomyxoviridae). A unique feature of nairoviruses in contrast to all other sNSVs is the size and composition of their L protein: it is nearly twice the size, and at the very N terminus, where in other L proteins the cap-snatching endonuclease is located, it harbors an ovarian tumor domain, which suppresses the cellular immune response (13). Using a virus-like particle system for CCHFV, the cap-snatching endonuclease has been located near residue Asp^693^, but the exact domain boundaries remain unknown (14).

We succeeded in the recombinant expression and purification of the isolated putative cap-snatching endonuclease of three different nairoviruses: CCHFV, Erve virus (EREV) and Nairobi sheep disease virus (NSDV), and the phlebovirus Rift Valley fever virus (RVFV). A recent comparative study classified the sNSV endonucleases into two functionally distinct groups, which—dependent on the presence or absence of a histidine residue in the active site—were named His+ and His− endonucleases (10). So far, all currently known endonucleases from Arenaviridae belong to the His−, and all endonucleases from Bunyavirales and Orthomyxoviridae belong to the His+ group.

Based on our biochemical and mutational studies, we propose to assign the nairovirus endonuclease into the group of His− endonucleases with similarities to His+ endonucleases, presenting a unique active site conformation. Solution scattering data showed that despite a much larger overall size of more than 300 residues compared with approximately 200 residues for related endonucleases, the overall shape of the domain is highly similar indicating structural conservation. Based on our data we propose a structural topology of the putative CCHFV cap-snatching endonuclease and evolutionarily position the nairovirus cap-snatching endonuclease in between the Arenaviridae and Bunyavirales.

## Results

### Identification of domain borders of the CCHFV endonuclease domain

**Figure 1.**
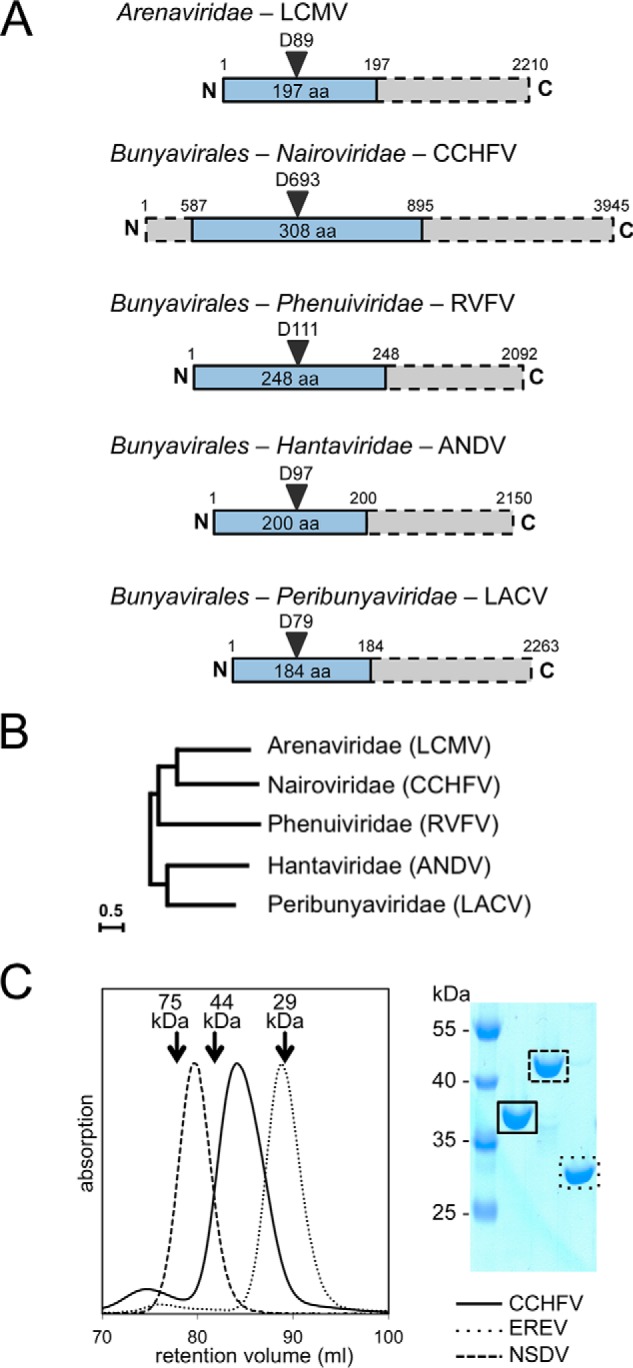
**Domain boundaries of the CCHFV cap-snatching endonuclease.** *A*, schematic diagram of cap-snatching endonuclease domains (*blue*) within L proteins (*gray bars*) of sNSVs. The order/family, an example species, and residue numbers for the example species are indicated. *B*, evolutionary relationship between sNSVs based on the RdRp sequences, displayed in an unrooted maximum likelihood tree (adapted from Ref. 15), shows close relationship between Nairoviridae and Arenaviridae. *C*, size-exclusion chromatography and Coomassie-stained denaturing gel show that the three nairovirus endonuclease domains are pure, monomeric, and of different sizes. The molecular masses and retention volumes of size standard proteins are indicated *above* the chromatogram.

The domain arrangement within L proteins varies dramatically between Nairoviridae and other members of the Bunyavirales and Arenaviridae (Fig. 1*A*), which complicates the localization of their cap-snatching endonuclease. From phylogenetic analysis based on the RdRp domain within the L protein (Fig. 1*B*, adapted from Ref. 15), Nairoviridae can be evolutionarily allocated between Arenaviridae and Phenuiviridae. Using their virus-like particle system for CCHFV, Devignot *et al.* (14) could identify the active site Asp of the cap-snatching endonuclease as residue Asp^693^. To identify the domain boundaries and produce the isolated endonuclease protein domain, we aligned several nairovirus L protein sequences, using only the residue range between approximately 400 and 1,200 and the secondary structure guided multiple sequence alignment tool PRALINE (16) (Fig. S1). Keeping predicted secondary structure elements intact, we proposed potential N and C termini for the endonuclease domain, ranging from residues 513 to 629 as the N-terminal border and from residues 854 to 1,059 as the C-terminal border (Fig. S2). Constructs were amplified from the CCHFV gene (Uniprot D4NYK2), cloned with an N-terminal, cleavable histidine tag, and tested for soluble expression in *Escherichia coli*. Only one construct, composed of residues 587–895, was successfully expressed with high yields and could be purified to homogeneity (Fig. 1*C*). The sequence alignment shows an extremely variable region starting near residue 750 (Fig. S1). In analogy to the CCHFV domain, we expressed and purified two additional endonuclease domains: from EREV (Uniprot J3RTH4), which is the smallest of all known homologs; and from NSDV (Uniprot G0X446), which has an additional 82 residues in the variable region, compared with EREV. Size-exclusion chromatography of the purified domains confirmed the presence of monomeric and pure protein in all three cases (Fig. 1*C*).

### Expression of related cap-snatching endonucleases

For comparative studies of endonucleases from a diverse set of sNSVs, we expressed and purified the arenavirus endonuclease from LCMV (8), the hantavirus endonuclease from ANDV (9), and the peribunyavirus endonuclease from LACV (11), as described. To better cover the families of Bunyavirales, we included the endonuclease from the phenuivirus RVFV. Using a reverse genetics system, the position of its active site had been proposed near residue Asp^111^ (17). We cloned and tested N-terminal L protein fragments from the L protein of RVFV strain ZH501 (Uniprot A2SZS6), ranging from 174 to 387 residues in length (Fig. S2). The highest yield of soluble protein was obtained for the construct ending at position 248. Pure RVFV endonuclease was produced using nickel affinity and size-exclusion chromatography. In addition to the WT endonuclease domains, enzymatically inactive mutants were prepared by mutating the catalytic aspartate from the PD(E/D)K motif to alanine (D111A).

### In vitro nuclease activities of cap-snatching endonucleases

**Figure 2.**
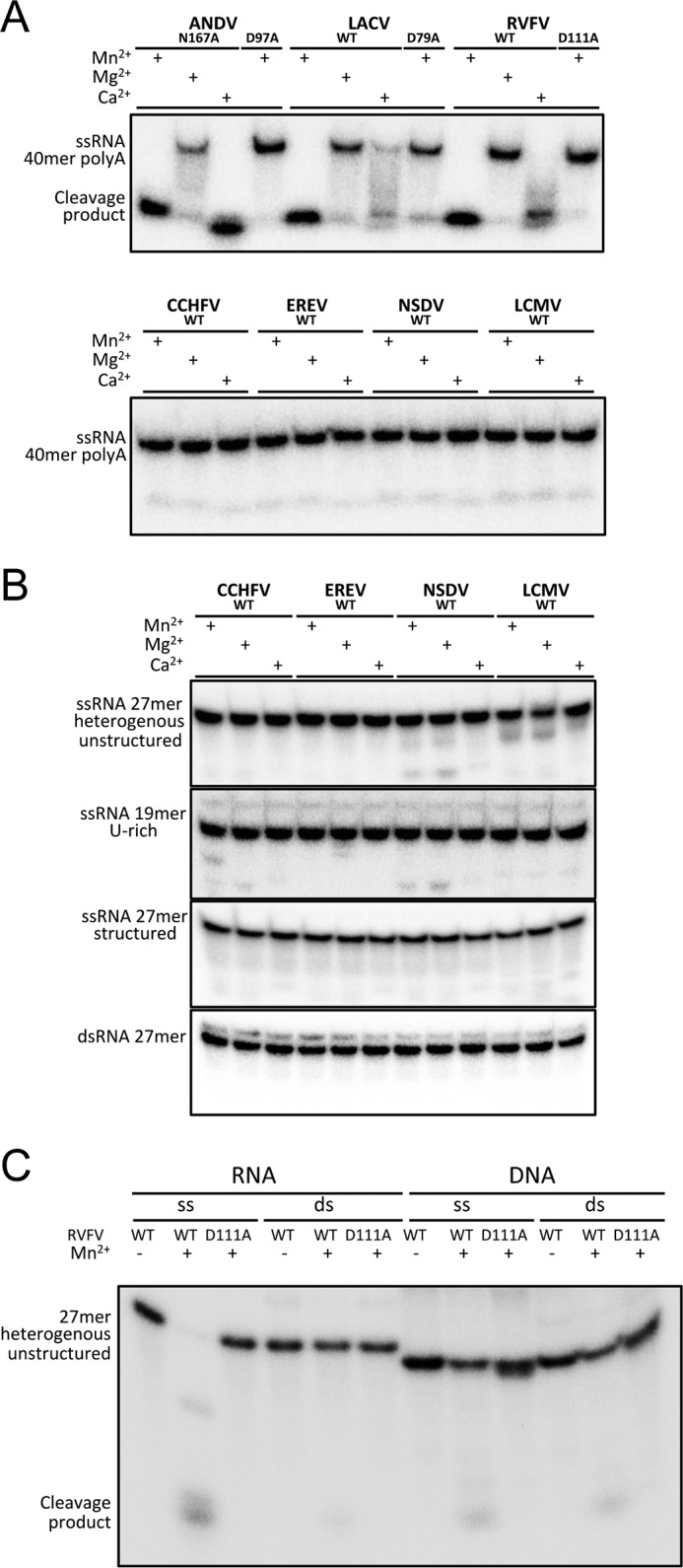
**Nuclease activity of different cap-snatching endonucleases.** Enzymes were incubated with a radioactively labeled nucleic acid substrate in the presence or absence of divalent metal ions, as indicated. Substrate and reaction products were separated on a denaturing polyacrylamide gel and visualized by autoradiography. *A*, His+ endonucleases show activity on ssRNA in the presence of manganese and calcium, whereas their catalytic site mutants are inactive. *A* and *B*, all three nairovirus endonucleases, as well as the His− endonuclease from LCMV, do not show significant activity on any of the tested RNA substrates. *C*, all tested His+ endonucleases (ANDV, LACV, and RVFV) do not cleave dsRNA, ssDNA, or dsDNA, but only ssRNA as shown exemplarily for the RVFV endonuclease.

Divalent cation–dependent nuclease activity has been described for the isolated domains of four His+ endonucleases: the influenza virus PA subunit, two endonucleases from closely related hantaviruses (ANDV and HNTV), and one from the peribunyavirus LACV. The available data for His− endonucleases from arenaviruses is inconclusive: weak activity was shown for the endonucleases from LCMV and Lassa virus (8, 18), but a more recent and thorough study could detect basically no activity for both proteins (10). We performed nucleic acid degradation experiments with our set of seven endonuclease domains from LCMV, LACV, ANDV, RVFV, CCHFV, NSDV, and EREV (Fig. 2*A*). The reactions were carried out at 30 °C for 60 min with 2 mm of divalent metal ion (Mg^2+^, Mn^2+^, Ca^2+^), 0.1 μm nucleic acid substrate, and 1 μm protein. As expected, ANDV and LACV show the highest nuclease activity on ssRNA (structured and unstructured) as substrate in the presence of Mn^2+^ and lower activity with Ca^2+^. LCMV does not show significant activity under any of the tested conditions. RVFV, which from sequence alignments with LACV and ANDV could be assigned to be a His+ endonuclease (Fig. S3), showed the expected activity on ssRNA in the presence of Mn^2+^. To verify the classification of the RVFV domain as His+ endonuclease, we mutated the predicted His to Ala (H79A), and indeed we observed very low residual catalytic activity as well as strongly reduced thermal stabilization by manganese ions for the mutant (Fig. S4).

Merely based on the amino acid composition, the nairovirus endonucleases cannot be assigned to a family of endonucleases. Neither the CCHFV, EREV, nor NSDV endonucleases showed enzymatic activity on any of the tested substrates: structured *versus* unstructured ssRNA, poly(A) RNA, U-rich RNA, and double-stranded (ds) RNA (Fig. 2*B*) behaving like the LCMV His− endonuclease. Also single and dsDNA substrates could not be cleaved by any of the tested endonucleases (shown exemplarily for the RVFV endonuclease in Fig. 2*C*). To rule out the possibility of sequence specificity, we performed the assay using a long *in vitro* transcribed 470-nucleotide RNA molecule with heterogeneous sequence, but again no nuclease activity could be detected for either LCMV or the nairovirus endonuclease domains (Fig. S5).

### Thermal stability of WT cap-snatching endonucleases

**Figure 3.**
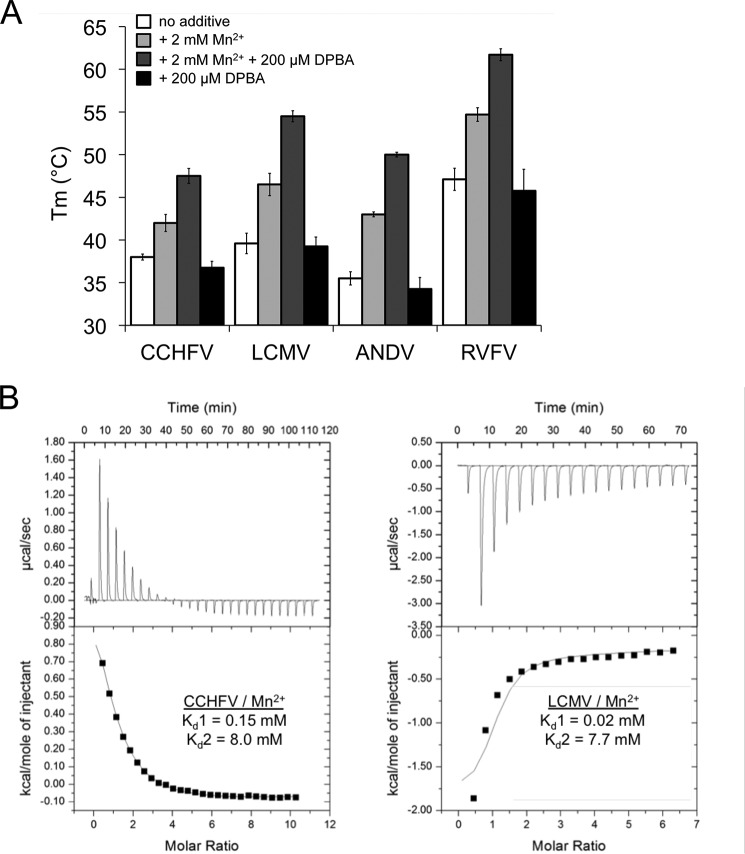
**Stabilization of cap-snatching endonucleases by a known endonuclease inhibitor and manganese binding.** *A*, thermal stability of His+ and His− endonucleases in the presence of 2 mm MnCl_2_ and 200 μm DPBA measured in a thermal shift assay (*T*_m_, melting temperature). Thermal stabilization by DPBA is only observed in the presence of manganese ions. *B*, manganese binding to 160 μm CCHFV (*left panel*) or 140 μm LCMV (*right panel*) endonuclease protein was measured at 20 °C by isothermal titration calorimetry. The *upper plot* shows the binding isotherm, and the *lower plot* shows the integrated values. The dissociation constants from fitting a two-site sequential binding mode (*K_d_*_1_ and *K_d_*_2_) to one representative experiment are indicated.

Without any detectable activity for the CCHFV L protein domain, we set out to find an alternative approach to confirm the existence of an endonuclease active site in our isolated domain. The cap-snatching endonucleases from LCMV and Lassa virus as well as from influenza virus, LACV, and both studied hantaviruses bind to manganese ions and to the influenza virus endonuclease inhibitor 2,4-dioxo-4-phenylbutanoic acid (DPBA) (19), as shown by an increase in thermal stability upon binding (9–11). As described for related endonucleases, binding of DPBA to the CCHFV domain is only observed in the presence of manganese ions. For the His+ endonucleases with detectable *in vitro* activity (influenza virus, LACV, ANDV, and HNTV), an inhibitory effect of DPBA could be shown with IC_50_ values in the range of 5–50 μm (9, 10, 20). To support the hypothesis that the isolated domain of the CCHFV L protein indeed contains an active site similar to the active sites of the known cap-snatching endonucleases, we measured the melting temperature (*T*_m_) increase by a thermal shift assay in the presence of 2 mm Mn^2+^ alone and with additional 200 μm DPBA (Fig. 3*A*). Although the detected thermal shift upon addition of metal ions and inhibitor is slightly lower for CCHFV than for LCMV, ANDV, and RVFV (10 °C *versus* 14–15 °C), the stabilizing effect is significant. Therefore, we propose the existence of a metal- and DPBA-binding endonuclease active site for CCHFV. To verify these findings we measured Mn^2+^ binding to the CCHFV and LCMV endonucleases by ITC (Fig. 3*B*). We find that binding of Mn^2+^ ions to the LCMV endonuclease is exothermic at 20 °C, as was described for the closely related Lassa virus endonuclease (10), whereas binding to the CCHFV endonuclease is endothermic under the same conditions. The dissociation constants *K_d_* for metal ion binding are obtained by model fitting, in which a cooperative binding mode with two-step sequential binding was used, in analogy to the published binding curves for LCMV and Lassa virus (10). The obtained *K_d_* values for LCMV are comparable with the published values for the closely related Lassa virus (10), and the affinity to the first manganese ion is approximately seven times higher than for CCHFV. This is in accordance with the lower thermal shift detected for CCHFV in contrast to the other endonucleases.

### Divalent cation coordinating residues in the active site of CCHFV endonuclease

**Figure 4.**
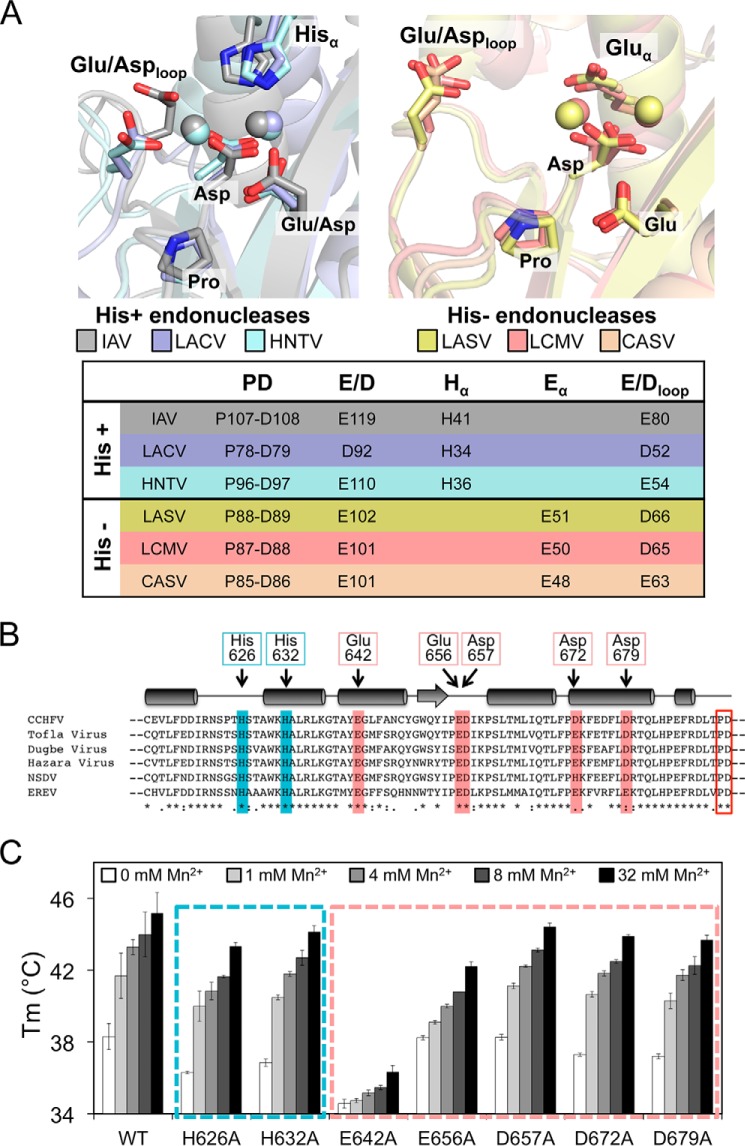
**CCHFV endonuclease active site conformation.** *A*, active site architecture of His+ (*left panel*) and His− (*right panel*) endonucleases. Superposition of relevant side chains (residue numbers are given in the table below) and the divalent metal ions of IAV, LACV, and HNTV are opposed to LASV, LCMV, and CASV. *B*, sequence alignment of the relevant area in the endonuclease domain of nairoviruses and potential His or Glu/Asp residues chosen for mutational studies. The alignment was generated using ClustalΩ (43). Conservation between residues is indicated as follows: *, identical; :, highly similar; ., similar. The predicted secondary structure elements are shown as *cylinders* for α-helices and *arrows* for β-sheets. Residues tested in mutagenesis studies are marked in *blue* (His) and *red* (Glu/Asp). The PD motif of the active site is shown at the *far right. C*, a Glu side chain but none of the His are involved in metal ion coordination, as shown by thermal stability testing of different CCHFV single-site mutants in presence of increasing amounts of Mn^2+^. As in *B*, His mutants are highlighted with a *blue frame*, and Glu/Asp mutants are highlighted with a *red frame. IAV*, influenza A virus; *LASV*, Lassa virus; *LCMV*, lymphocytic choriomeningitis virus; *CASV*, California Academy of Sciences virus.

Fig. 4*A* summarizes the central chemical differences between metal ion coordination in His+ and His− endonucleases: all of them contain the three active site residues Pro, Asp, and Glu/Asp from the PD(E/D)K motif. The His+ endonucleases possess the additional name-giving histidine to coordinate the first metal ion. A conserved Asp in a loop close to the active site was shown to be involved in the coordination of the second metal ion and essential for catalytic activity. The His− endonucleases in contrast have a Glu, which is located in the same α-helix as the His in the His+ endonucleases. This residue is also involved in metal ion coordination but from structural data seems to interact with the second metal ion. All endonucleases possess a conserved Glu/Asp in a flexible loop close to the active site. In His+ endonucleases this residue is clearly coordinating the second metal ion, whereas in His− endonucleases it is too far away to be involved in metal ion coordination and was indeed shown to be dispensable for viral transcription (10).

Sequence alignment between the CCHFV endonuclease domain and either the His+ or the His− endonucleases is barely possible. In addition to the Pro and Asp from the PD(E/D) motif, no reliable conclusion can be drawn. Therefore, we designed a set of single-site mutants to identify the missing residues with a role in metal ion coordination in the area preceding the PD(E/D)K motif (Fig. 4*B*): two His and five Glu/Asp residues are conserved among nairovirus endonuclease domains and were mutated to Ala. The thermally stabilizing effect of manganese ions on the proteins was tested for all seven mutants compared with the WT protein (Fig. 4*C*). The results show that none of the histidines seem to play a role in metal ion coordination, whereas one of the Glu mutants (E642A) shows a clear reduction in thermal stabilization by manganese. Interestingly this Glu lies in a predicted α-helix, just as the Glu found in His− endonucleases. At this point we therefore concluded that the CCHFV cap-snatching endonuclease belongs to the His− endonucleases.

### Structural analysis of CCHFV endonuclease domain

**Figure 5.**
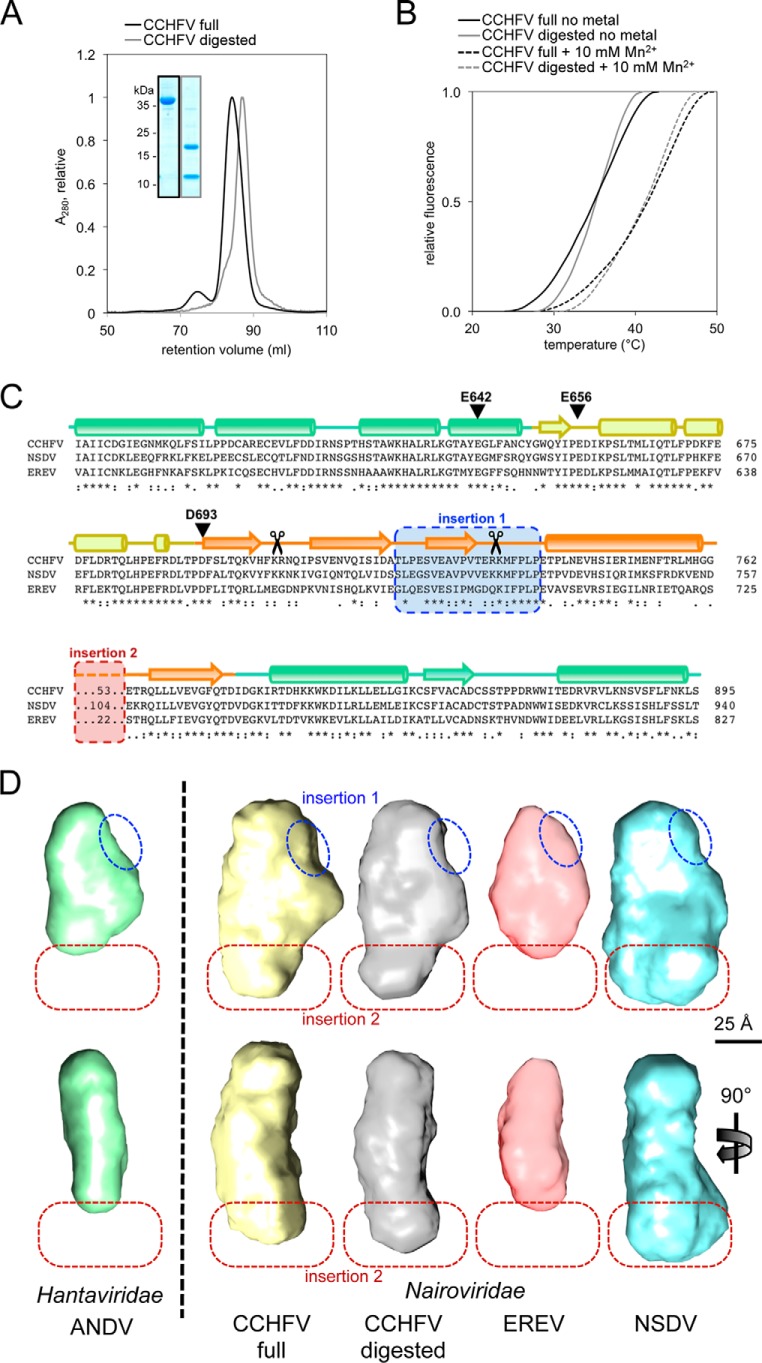
**Structural analysis of the CCHFV endonuclease and identification of two insertions.** *A*, elution profiles from the size-exclusion column show a smaller size for the CCHFV endonuclease domain after trypsin treatment (*gray*) compared with the nontreated domain (*black*). *Inset*, the peak fractions are analyzed on a Coomassie-stained denaturing gel. *B*, melting curves of digested (*gray*) and full (*black*) CCHFV endonuclease domains are shown in absence (*solid line*) or in presence of 10 mm Mn^2+^ (*dashed line*). *C*, the structure-based sequence alignment of the three analyzed nairoviruses CCHFV, NSDV, and EREV was generated by PRALINE (16). Predicted and conserved secondary structure elements are shown as *cylinders* for α-helices and *arrows* for β-sheets. The color coding is as in Fig. 6. Insertion 1 (*blue*) is missing in the digested CCHFV domain (trypsin cleavage sites as determined by MS are indicated with *scissors*), and insertion 2 (*red*) is of variable size in the three different proteins. *D*, *ab initio* shapes of three nairovirus endonucleases (CCHFV, EREV, and NSDV) and the digested CCHFV domain compared with the ANDV endonuclease domain (9) as example for a non-nairovirus homolog are shown in front view (*top panel*) and side view (*bottom panel*). As in *C*, the proposed locations of the two insertions are marked with *blue* and *red dashed lines*.

The nairovirus cap-snatching endonuclease domain is significantly larger than the respective domains of other related viruses. To identify structural differences and similarities, we compared a set of four different proteins: the ANDV endonuclease domain as representative of a smaller non-nairovirus endonuclease and the three nairovirus endonucleases from CCHFV, EREV, and NSDV. In addition, we performed limited proteolysis with the CCHFV endonuclease (Fig. S6). The addition of low amounts of trypsin resulted in a protein domain, which consisted of two peptide chains, as seen on a denaturing gel (Fig. 5*A*). The fragments co-eluted from the size-exclusion chromatography later than the undigested domain, indicating a smaller size. Thermal stabilization by manganese ions was basically identical for the digested and the undigested protein domain, indicating the presence of a structurally intact metal coordination site even after digestion (Fig. 5*B*).

To further analyze the composition of this digested, heterodimeric complex, we performed native MS analysis with the undigested and trypsin-treated CCHFV domain (21, 22). Examination of the undigested domain shows the presence of a monomeric protein with a mass of 37,498 ± 5 Da corresponding to the calculated molecular mass of the endonuclease domain assuming the N-terminal methionine was processed. To a small extent, homodimers and trimers were also detected (Fig. S7*A*). In contrast, we detect a lower molecular mass of 34,430 ± 50 Da for the digested protein. (Fig. S7*B*). MS/MS experiments at higher collision energies revealed that the 34-kDa species indeed constitutes a heterodimeric protein complex formed by the N- and C-terminal domain (Fig. S7*C*). The smaller subunit has a mass of 15,729 ± 1 Da, and the larger subunit comes in two masses of 18,667 ± 2 and 18,539 ± 1 Da.

Comparing molecular masses of the nontreated domain and the summed fragment masses of the treated equivalent identifies a mass loss of 3,102/3,230 Da after trypsin digestion. The resulting mass difference can be clearly assigned to an excised peptide delimited by two trypsin cleavage sites: Arg^704^ and Arg^733^/Lys^734^ (indicated with *scissors* in Fig. 5*C*). The presence of two different C-terminal subunit species are the result of two adjacent protease cleavage sites: Arg^733^ and Lys^734^, which is further evidence for the identity of the peptide (Fig. S7, *C* and *D*).

The region indicated as *insertion 1* in Fig. 5*C*, which is conserved in nairoviruses, is part of the cleaved-out peptide. The region denoted as *insertion 2* is not conserved but of variable size and sequence. We propose that the smaller non-nairovirus endonucleases, such as ANDV, are missing both insertions.

To prove this theory and obtain structural data on the global shape of the endonucleases, we performed SAXS studies with all five proteins. The solution structures from *ab initio* modeling based on the scattering curves are shown in Fig. 5*D* (for scattering curves see Fig. S8). All proteins have a similar elongated and flat shape but differ in their overall dimensions. Comparison of the structures allows for the identification of both insertions: insertion 1, which is missing in the digested CCHFV protein and in the ANDV endonuclease is clearly visible in all three nondigested nairovirus proteins (*blue dashed line* in Fig. 5*D*); and insertion 2, which is equally small in EREV and ANDV, is clearly visible in the CCHFV structure and even larger in the NSDV structure and is located on the opposite side of insertion 1 (*red dashed line* in Fig. 5*D*). To check the plausibility of the SAXS models, we set out to generate a homology-based structure model of the putative CCHFV endonuclease domain to fit it into the respective SAXS envelope. Because of the lack of structural information from homologous proteins, several insertions cannot be predicted correctly. Some of the obtained models for the core of the CCHFV putative endonuclease are shown in Fig. S9. Although this core region is more reliably predicted than the insertions, the variability is significant. The insertions are only drawn schematically to avoid the display of artificial modeling results. Nevertheless, the positions of these insertions nicely coincide with the additional electron density seen in the SAXS envelope, providing further evidence to the SAXS models.

### Protein topology of the CCHFV cap-snatching endonuclease

**Figure 6.**
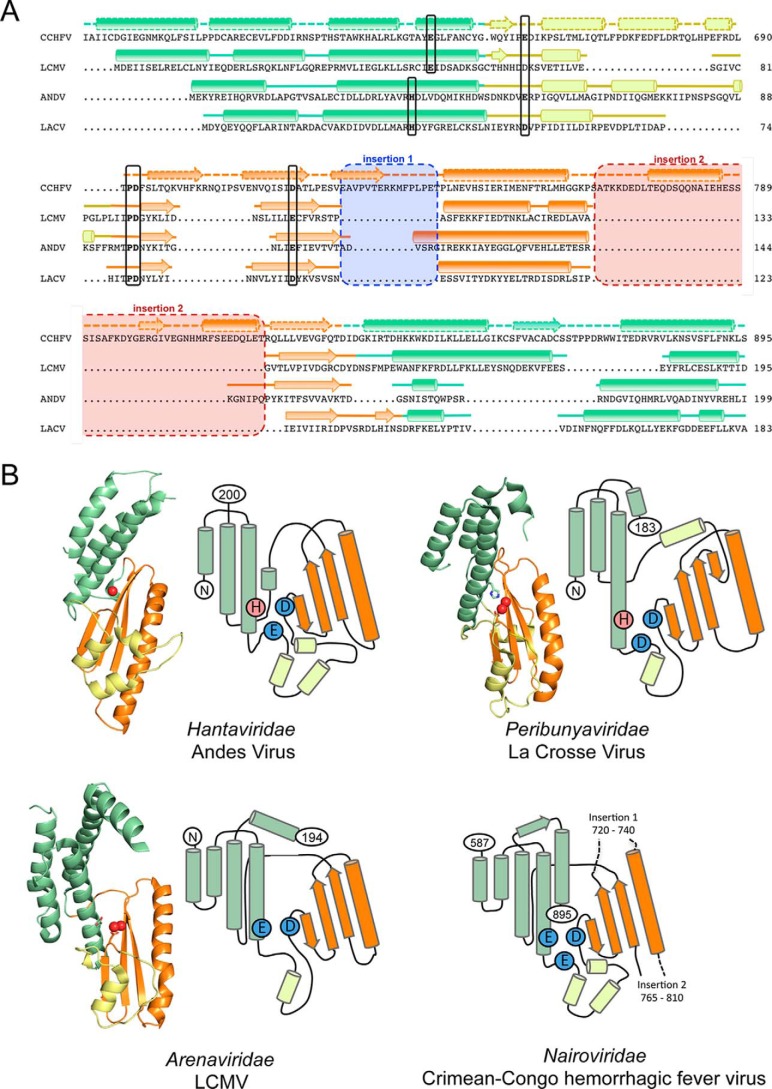
**Proposed structural topology of the putative CCHFV cap-snatching endonuclease.** *A*, the sequence of the CCHFV endonuclease was manually aligned with His+ and His− endonucleases for which atomic structures are available. Secondary structure elements are shown as *cylinders* for α-helices and *arrows* for β-sheets and were taken from secondary structure prediction for CCHFV and from the crystal structures for the other proteins. Residues with a role in metal ion coordination are highlighted and align with the corresponding residues in the related proteins. Insertions 1 and 2, which are only present in nairoviruses, are highlighted in *blue* and *red*, respectively. *B*, structural topology of two His+ and a His− endonuclease (based on their crystal structures) and predicted topology of the CCHFV endonuclease, which takes an intermediate position. Important metal ion–coordinating side chains in the active site are shown as *red circles* (His) and *blue circles* (Glu/Asp). Related structural elements are colored as in *A*.

Based on our biochemical, mutational, and structural data we set out to propose a protein topology for the putative CCHFV cap-snatching endonuclease. Therefore, we first created a structure-based sequence alignment between the CCHFV (using predicted secondary structure elements), LCMV (PDB code 5LTF), ANDV (PDB code 5HSB), and LACV (PDB code 2XI5) proteins (Fig. 6*A*). The secondary structure elements are colored according to their structural role in the endonuclease: *green*, helix bundle formed by α-helices from the N and C terminus of the domain; *yellow*, loop-rich region between the helix bundle and the active site; and *orange*, central region formed mainly by a large α-helix and β-sheet. The residues involved in the coordination of metal ions are marked and convincingly align. The two insertions described above are indicated. Fig. 6*B* shows the protein topology for the LCMV, ANDV, and LACV endonucleases next to their crystal structures. Central active side residues are marked: the catalytic Asp, the His of the His+ endonucleases, the Glu of the His− endonucleases, and the additional Glu/Asp in the loop region close to the active site, which only plays a role in metal ion coordination in the His+ endonucleases. Interestingly one of the Glu residues (Glu^656^), which we had tested in our mutational studies (Fig. 4*C*) and which showed a slightly decreased thermal stabilization by manganese, aligns with the Glu/Asp in this loop (Fig. 6*A*). To undermine these data, we performed ITC measurements with the CCHFV E656A mutant and indeed barely any manganese binding could be detected (Fig. S10). All studies to date suggested that only His+ endonucleases use this acidic residue for the coordination of their second metal ion. The CCHFV endonuclease seems to take a unique position in between the currently known His+ and His− endonucleases, because features of both enzyme classes are present in its active site: On the one hand, it contains an essential Glu instead of a His, like His− endonucleases. On the other hand, the Glu/Asp residue in the loop region is involved in metal ion coordination, like in His+ endonucleases.

## Discussion

A large set of important human pathogens belongs to the group of sNSVs, which use the unique cap-snatching mechanism for transcription initiation. This includes well-studied viruses like the influenza virus and the under-researched and deadly Crimean–Congo hemorrhagic fever virus (CCHFV). Nairoviridae in the order of Bunyavirales, including its most famous member, CCHFV, take an exceptional position within the sNSVs, because their L protein is almost twice the size of all other L proteins. The only reliably predictable domain is the RdRp domain, which is of similar size in all L proteins. It is currently unknown why the L proteins of nairoviruses evolved differently and what the role of most of the additional regions is. In contrast to the smaller L proteins, which contain the cap-snatching endonuclease at the very N terminus with the active site Asp located near residues 80–110, the catalytic Asp of the CCHFV endonuclease was proposed to be Asp^693^ (14). Instead, the first ∼160 residues of the nairovirus L protein form an ovarian tumor domain, which is involved in the suppression of the innate immune response (13). We identified the domain boundaries of the putative nairovirus cap-snatching endonuclease and recombinantly produced the protein from CCHFV, EREV, and NSDV, with lengths of 308, 277, and 359 residues, respectively. This variation within the endonuclease domain is surprising, because the corresponding domains are very conserved within other virus families. To obtain a more complete picture of the enzyme family of cap-snatching endonucleases, we also expressed and purified for the first time the first 248 residues of the L protein from RVFV (Phenuiviridae). This domain contains an active endonuclease, as proposed. Also, we included already described endonucleases from LCMV (Arenaviridae), LACV (Peribunyaviridae), and ANDV (Hantaviridae) in our study.

Our new data further establish the classification of cap-snatching endonucleases into His+ and His− endonucleases, dependent on the presence of a metal ion–coordinating His in the active site, as was proposed by Reguera *et al.* (10). As expected, the RVFV endonuclease belongs to the His+ endonucleases, and its catalytic activity is similar to other related enzymes. Mutation of the metal ion–coordinating His abolishes both thermal stabilization by manganese as well as nuclease activity. In contrast, we could not detect nuclease activity for any of the nairovirus endonucleases *in vitro*. The same is true for our prototypic His− endonuclease from LCMV: under the same conditions where all tested His+ endonucleases show clear activity on ssRNA, no nuclease activity could be observed for the LCMV enzyme. Indeed, mutational studies showed that no His is involved in metal ion coordination in the CCHFV enzyme. This is in accordance with the hypothesis from Reguera *et al.* (10), who proposed that the existence of a His in the active site is needed for strong and clearly detectable enzymatic *in vitro* activity. It is still unclear why the isolated His− endonucleases possess such a reduced activity. It could be much lower and more tightly regulated also in the full viruses and just below the detection limit of our assay. It is equally conceivable, however, that His− endonucleases need an additional co-factor or an activating protein to reach enzymatic activities in the range of the His+ endonucleases.

From the available data so far, it was proposed that all His+ endonucleases use an additional conserved acidic residue in a loop region to coordinate the second metal ion in the active site (11, 20). Although a similar residue is also present in the corresponding loop region in His− endonucleases, no role in metal ion coordination or transcription initiation could be assigned to this residue (10). The presented data led to the conclusion that the CCHFV endonuclease takes an intermediate position between the His+ and His− endonucleases: even in the absence of a metal ion–coordinating His in the active site, an additional Glu is clearly involved in metal ion binding. From structure-based sequence alignments, we propose that this residue is located in the same loop region as the corresponding residues in His+ endonucleases. These findings nicely fit to phylogenetic analyses based on the sequence of the RdRp, where the Nairoviridae cluster with the Arenaviridae (23) and occupy an intermediate position between Bunyavirales and Arenaviridae. It was even recently proposed to consider Arenaviridae in future taxonomic changes as a possible family or genus related to Nairo-like Bunyaviruses (24).

The cap-snatching endonuclease of influenza virus has been proven to be a valuable drug target (25). Biochemical and structural data of different cap-snatching endonucleases with DPBA (PDB code 5LTN; Refs. 11, 26, and 27) indicate that potential drugs could work as broad-spectrum antivirals against a large set of human pathogens. To further exploit this viral mechanism for therapeutic applications, we need to widen our knowledge on this class of enzymes. For high-throughput screening applications recombinantly expressed proteins from different cap-snatching endonucleases are needed, preferably from all virus families of sNSV that contain human pathogens. Therefore, our new data on the cap-snatching endonuclease from the phenuivirus RVFV and the putative endonuclease from the nairovirus CCHFV, which both are on the list of under-researched pathogens of the World Health Organization R&D blueprint from 2018 (28), present an important progress for the future development of antivirals against sNSV infections.

## Experimental procedures

### Cloning, expression, and purification

Based on bioinformatics analysis of the primary sequence CCHFV L protein fragments of different lengths harboring the putative endonuclease domain were designed. The sequences were amplified by PCR using synthesized L gene sequences of CCHFV, EREV, and NSDV (GenBank^TM^ accession nos. ADD64466.1, AED88229.1, and AFH89032.1) as templates. Sequences were cloned into pOPIN-F (N-terminal His tag with 3C protease cleavage site) or pOPIN-J (N-terminal His tag plus GSH *S*-transferase with 3C protease cleavage site) using the In-Fusion HD EcoDry cloning kit (Clontech) (29). L protein mutants were generated via a classical two-step PCR mutagenesis approach as described previously (30).

The CCHFV and NSDV endonuclease proteins were expressed with an N-terminal His tag and a 3C protease cleavage site in *E. coli* strain BL21 (DE3) in TB medium with 100 μm carbenicillin at 17 °C overnight after induction with 0.5 mm isopropyl β-d-thiogalactopyranoside. After pelleting, the cells were resuspended in lysis buffer containing 80 mm sodium phosphate, pH 6.8, 1 m NaCl, 5% glycerol, 10 mm imidazole, and 0.1 mm PMSF. Cells were disrupted by sonication, and the native proteins were purified by nickel affinity chromatography from the soluble fraction after centrifugation. The proteins were eluted in buffer containing 50 mm Tris, pH 7.5, 250 mm NaCl, 10% glycerol, and 250 mm imidazole. The eluted fractions were pooled and after dilution loaded on a Source 15Q column. Anion-exchange chromatography was performed by salt gradient with buffer A containing 20 mm Tris, pH 7.5, 80 mm NaCl, and 5% glycerol and buffer B containing 20 mm Tris, pH 7.5, 1 m NaCl, and 5% glycerol. Eluted fractions were pooled, and the His tag was removed by incubation with GST-tagged 3C protease at room temperature for 4 days (CCHFV) or overnight (NSDV). The protease was removed by GSH-Sepharose.

The EREV endonuclease protein was expressed with N-terminal GST tag and 3C cleavage site as described above. Cell pellets were resuspended in lysis buffer containing 50 mm Tris, pH 7.5, 1 m NaCl, 5% glycerol, and 0.1 mm PMSF, and the protein was purified by GSH-Sepharose and on-column cleavage with GST-tagged 3C protease for 4 h. The protein was recovered from the Sepharose with a buffer containing 20 mm citrate, pH 6.0, and further purified by cation exchange chromatography with a HiTrap heparin column and a constant NaCl gradient up to 1 m.

RVFV endonuclease protein was expressed with N-terminal His tag and 3C cleavage site as described above. The cells were resuspended in a buffer containing 50 mm HEPES, pH 7.5, 300 mm NaCl, 5% glycerol, 1 mm DTT, and 0.1 mm PMSF. The protein was purified by nickel affinity chromatography.

LCMV, LACV, and ANDV endonucleases were expressed and purified according to the literature (8, 9, 11). All proteins were finally purified by size-exclusion chromatography (Superdex 200 column; GE Healthcare) in a buffer containing 20 mm Tris, pH 7.5, 200 mm NaCl, and 5% glycerol. Purified proteins were concentrated using centrifugal filter devices, flash frozen in liquid nitrogen, and stored at −80 °C.

### Endonuclease assays

Nuclease activity was evaluated by incubating 20 pmol of endonuclease protein with 2 pmol of ^32^P-labeled single-stranded (ss) or dsRNA or DNA oligonucleotide substrates or *in vitro* transcribed RNA substrate in 20 μl of buffer containing 50 mm Tris, pH 7.5, 250 mm NaCl, 5% glycerol, and 0.5 unit/μl RNAsin (Promega) in the absence or presence of 2 mm MnCl_2_ at 30 °C for 1 h. The ssRNA 19-mer (5′-AUUUUGUUUUUAAUAUUUC-3′), ssRNA and ssDNA 27-mer (5′GA(U/T)GA(U/T)GC(U/T)A(U/T)CACCGCGC(U/T)CG(U/T)CG(U/T)C-3′), and poly(A) 40-mer oligonucleotides were obtained by gene synthesis (Biomers.net) and dissolved in RNase-free water. The *in vitro* transcribed RNA substrate (469 nucleotides) was transcribed by T7 RNA polymerase (T7 MegaScript) from 0.2 μg of PCR product according to the manufacturer's instructions in a 20-μl reaction from the pOPIN-M plasmid T7 promoter sequence. The 5′ ends of 100 pmol of each oligonucleotide substrate were radioactively labeled with 10 units of T4 polynucleotide kinase and 20 μCi of [γ-^32^P]ATP (Hartmann Analytics) in 20 μl of reaction volume for 1 h at 37 °C. Labeled oligonucleotides were separated from free [γ-^32^P]ATP by Illustra G-25 or G-50 MicroSpin columns (GE Healthcare). Labeled oligonucleotides were stored in RNase-free water in presence of 0.5 unit/μl RNAsin at 4 °C. Double-stranded oligonucleotides were obtained by annealing complementary ss 27-mer oligonucleotides by heating for 5 min at 98 °C followed by a slow cooling to 20 °C over 2 h.

The nuclease reaction was stopped by adding an equivalent volume of 2× loading buffer (98% formamide, 18 mm EDTA, 0.025 mm SDS, xylene cyanol, and bromphenol blue) and heating the samples at 98 °C for 5 min. The reaction products were separated by denaturing gel electrophoresis (7 m urea, 20% polyacrylamide, 90 mm Tris-HCl, 90 mm borate, 2 mm EDTA), and the gels were exposed to a Storage phosphor screen for 1 h at 4 °C and visualized by phosphor screen autoradiography scanning using a FLA 7000 Typhoon scanner (GE Healthcare).

### Thermal stability assay

The thermal stabilization of endonuclease proteins by manganese ions was measured by thermal shift assay (31). 0.1 mg/ml endonuclease WT or mutant was incubated in absence or presence of increasing MnCl_2_ concentrations (0.25–32 mm) and in the absence and presence of 200 μm of the endonuclease inhibitor DPBA in 25 μl of buffer containing 50 mm Tris, pH 7.5, 250 mm NaCl, 5% glycerol, and SYPRO orange (1:1,000). The increase of fluorescence signal was measured at a constant temperature gradient between 20 and 80 °C. The melting temperature was determined as the temperature where 50% of the protein is unfolded.

### Isothermal titration calorimetry

ITC measurements were performed at 20 °C using a VP-ITC calorimeter at the sample preparation and characterization (SPC) facility of the EMBL in Hamburg (32) with a sample cell containing 1.43 ml of 100–160 μm of CCHFV WT or E656A mutant endonuclease protein or 140 μm of LCMV WT protein. Titrations were made by injecting 15 μl of 5–8 mm manganese solution. All proteins were prepared for the measurement by excessive dialysis against buffer containing 50 mm Tris, pH 7.5, 150 mm NaCl, 2.5% glycerol at 4 °C. The ligand solution was prepared by dissolving MnCl_2_ dihydrate in dialysis buffer. The data have been evaluated using Origin 7 software (OriginLab).

### Small-angle X-ray scattering

SAXS analysis was performed with purified CCHFV, NSDV, and EREV endonuclease proteins at the EMBL P12 beamline of the PETRA III synchrotron storage ring at DESY (Deutsches Elektronen-Synchrotron) in Hamburg (33). Prior to SAXS analysis all proteins were purified by size-exclusion chromatography in buffer containing 50 mm Tris, pH 7.5, 200 mm NaCl, and 5% glycerol. Scattering intensity was measured at different protein concentrations between 0.5 and 5.0 mg/ml using a PILATUS 2M pixel detector at 3.1-m sample distance and 10 keV energy (λ = 1.24 Å) covering a momentum transfer range of 0.01 Å^−1^ < *q* < 0.45 Å^−1^ (*q* = 4π sinθ/λ, where 2θ is the scattering angle). All experiments were performed at 20 °C, and the data were processed as described (34). Scattering data were subtracted from the buffer signal and processed using the ATSAS 2.6 package (35). The forward scattering *I*(0) and the radius of gyration *R*_g_ were extracted from the Guinier approximation calculated with the AutoRG function within PRIMUS. The pair distribution function *P*(*r*) of the particle and the maximum size *D*_max_ were calculated with GNOM (36). *Ab initio* models were reconstituted with GASBORi (37). 30 independent GASBOR runs were performed, superimposed, and averaged using the program DAMAVER (38). Structures were visualized using UCSF Chimera (39).

### Native MS

Prior to native MS measurements, Micro Bio-Spin^TM^ 6 chromatography columns (molecular mass cut-off 6 kDa; Bio-Rad) were used to exchange purified protein samples to 250 mm ammonium acetate (99.99% purity; Sigma–Aldrich), pH 7.4, as buffer surrogate at 4 °C. Final concentrations for native MS analysis of the undigested CCHFV endonuclease domain and trypsin-digested equivalent were 10, 25, and 50 μm. Native MS experiments were carried out on a Q-TOF II mass spectrometer in positive electrospray ionization mode, which was modified to enable high mass experiments (40) (Waters and MS Vision). Sample ions were transferred into the vacuum by handmade capillaries at 10 mbar source pressure via a nano-electrospray ionization source. Borosilicate glass tubes (inner diameter, 0.68 mm; outer diameter, 1.2 mm; World Precision Instruments) were pulled into closed capillaries in a two-step program using a squared box filament (2.5 × 2.5 mm) within a micropipette puller (P-1000; Sutter Instruments). The capillaries were then gold-coated using a sputter coater (40 mA, 200 s, tooling factor of 2.3 and end bleed vacuum of 8 × 10–2 mbar argon; Q150R, Quorum Technologies Ltd.). Capillaries were opened directly on the sample cone of the instrument. Mass spectra in regular MS mode were obtained at a capillary voltage of 1.3 kV and a cone voltage of 150 V. To assign quaternary protein species, MS/MS experiments were performed using argon as collision gas (2.0 × 10–2 mbar). Here, collision voltage was varied between 5 V and 150 V. MS quadrupole profiles, and pusher settings were kept constant in all measurements to ensure comparability of results. For calibration of the data, a spectrum of cesium iodide was recorded on the same day of the particular measurement. The spectra were evaluated via MassLynx (Waters). Using this software, the peaks were assigned, and the respective masses were determined. The values of the shown averaged masses of the different species, as well as the corresponding standard deviation result from at least three independent measurements. Average full widths at half-maximum of each species are also reported (Fig. S7*D*). The narrow peak widths indicate rather homogenous samples except for the heterodimer.

### Molecular modeling

Molecular modeling was performed using the Bioinformatics Toolkit (41) and the program MODELLER (42). Prior to molecular modeling, secondary structure-based sequence alignments of the putative CCHFV endonuclease to either ANDV, LACV, or LCMV were created using HHPred followed by manual adjustments. To better evaluate the reliability of predicted models, different sets of input structures were used, and the resulting models were compared (Fig. S9*A*). This included the endonuclease domains from Arenaviridae (Lassa virus, PDB code 5J1N; Pichinde virus, PDB code 4I1T; LCMV, PDB code 5LTN; California Academy of Science virus, PDB code 5MV0), Hantaviridae (Andes virus, PDB code 5HSB; Hantaan virus, PDB code 5IZE), and Peribunyaviridae (La Crosse virus, PDB code 5XI7). Regions in which the predicted structures were extremely variable and did not contain any secondary structure elements are not shown in Fig. S9*A* and drawn schematically for one example in Fig. S9*B*.

## Author contributions

T. H., C. U., S. G., and S. R. conceptualization; T. H., J.-D. K., C. B., S. O., M. R., and C. U. investigation; M. R., C. U., and S. R. supervision; S. G. resources; S. G. and S. R. funding acquisition; S. R. methodology; S. R. writing-original draft.

## Supplementary Material

Supporting Information

## Footnotes

3 The abbreviations used are:

CCHFV: Crimean–Congo hemorrhagic fever virus
sNSV: segmented negative strand RNA virus
EREV: Erve virus
NSDV: Nairobi sheep disease virus
RVFV: Rift Valley fever virus
SAXS: small-angle X-ray scattering
PDB: Protein Data Bank
RdRp: RNA-dependent RNA polymerase
LCMV: lymphocytic choriomeningitis virus
ANDV: Andes virus
HNTV: Hantaan virus
LACV: La Crosse virus
DPBA: 2,4-dioxo-4-phenylbutanoic acid
PMSF: phenylmethylsulfonyl fluoride
ss: single-stranded
ds: double-stranded
SAXS: small-angle X-ray scattering.
