# Supplementary material for: Biochemical and structural studies reveal differences and commonalities among cap-snatching endonucleases from segmented negative-strand RNA viruses
Source: J Biol Chem. 2018 Oct 22;293(51):19686–98. doi: 10.1074/jbc.RA118.004373 (PMC6314124; doi:10.1074/jbc.RA118.004373)
Supplement: Supporting Information [file supp_293_51_19686__index.html]

Biochemical and structural studies reveal differences and commonalities among cap-snatching endonucleases from segmented negative-strand RNA viruses — Comparative study on cap-snatching endonucleases — Biochemical and structural studies reveal differences and commonalities among cap-snatching endonucleases from segmented negative-strand RNA viruses — Comparative study on cap-snatching endonucleases — Supporting Information 

# Biochemical and structural studies reveal differences and commonalities among cap-snatching endonucleases from segmented negative-strand RNA viruses

## Supporting Information

- Supporting Information (to be published online) - Supporting Figures S1 - S10
