# Supplementary material for: Biochemical and structural studies reveal differences and commonalities among cap-snatching endonucleases from segmented negative-strand RNA viruses
Source: J Biol Chem. 2018 Oct 22;293(51):19686–98. doi: 10.1074/jbc.RA118.004373 (PMC6314124; doi:10.1074/jbc.RA118.004373)
Supplement: Supporting Information [file supp_RA118.004373_138705_2_supp_222466_pgwcbb.pdf]

Supporting information

Biochemical and structural studies on the putative Crimean-Congo hemorrhagic fever virus cap-snatching endonuclease

Tobias Holm[1], Janine-Denise Kopicki[2], Carola Busch[1], Silke Olschewski[1], Maria Rosenthal[1], Charlotte Uetrecht[2,3], Stephan Günther[1] and Sophia Reindl[1,*]

Contents:

Figure S1
Sequence alignment of L protein sequences from different nairoviruses.

Figure S2
Constructs tested for soluble expression in *Escherichia coli* for (A) the CCHFV endonuclease and (B) the RVFV endonuclease.

Figure S3
Secondary structure based sequence alignment of endonuclease sequences from viruses of the *Phenui*-, *Hanta*- and *Peribunyaviridae* families.

Figure S4
The RVFV endonuclease belongs to the class of His+ endonucleases.

Figure S5
Comparison of IVT substrate degradation by 5 different endonuclease proteins of the order *Bunyavirales*.

Figure S6
Limited Proteolysis of CCHFV endonuclease protein and fragment characterization.

Figure S7
Trypsin digestion of the CCHFV endonuclease domain monitored by native MS.

Figure S8
SAXS scattering curves of nairovirus endonuclease proteins.

Figure S9
Homology-based structural models of the core of the CCHFV putative endonuclease structure and comparison to the SAXS envelope.

Figure S10
Affinity to manganese ions is strongly reduced in the CCHFV E656A mutant compared to the wild type.

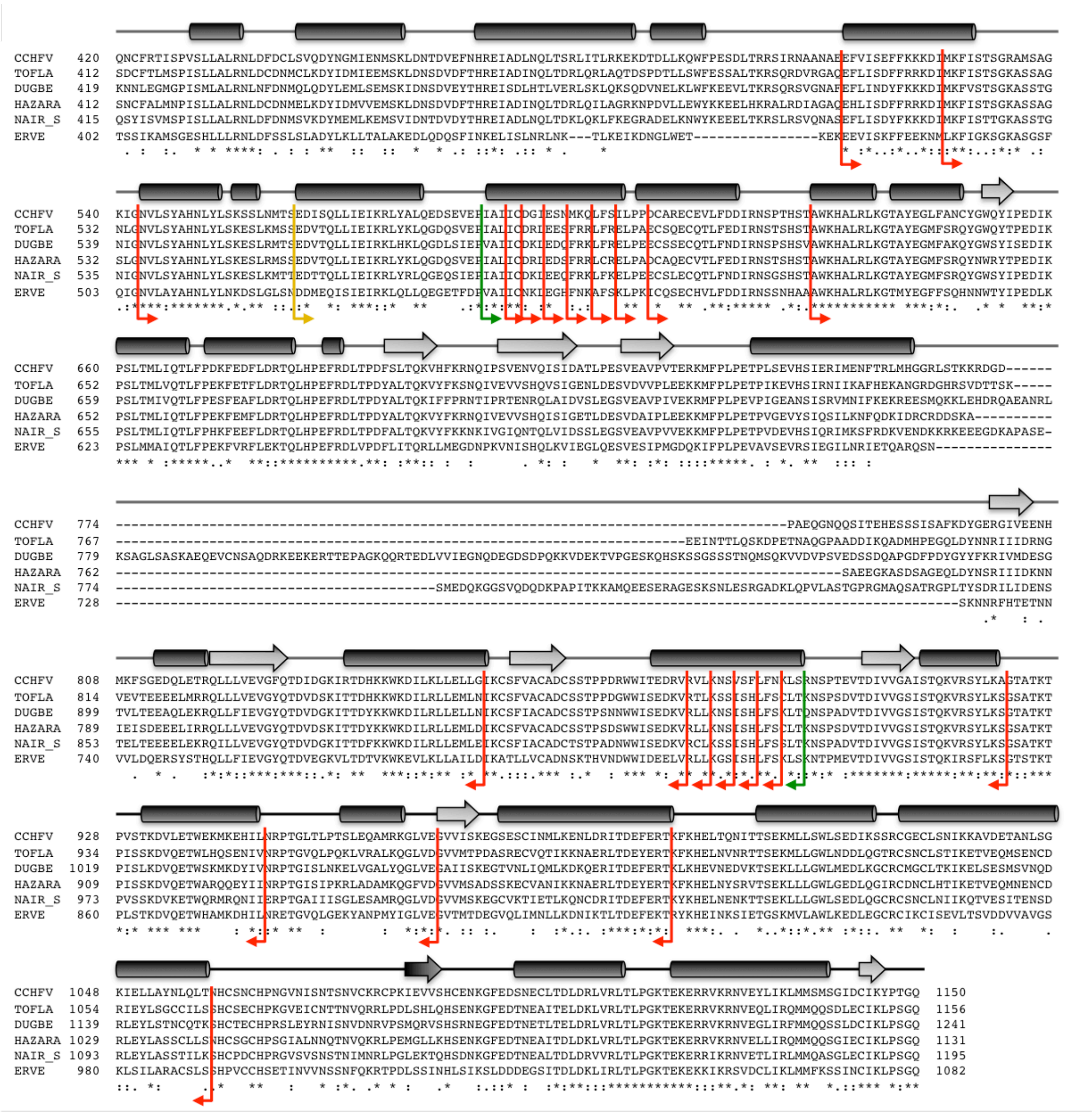

Figure S1. Sequence alignment of L protein sequences from different nairoviruses. Only the residue range from ~400 - 1150 was used. Predicted secondary structure elements are shown as cylinders for α-helices and arrows for β-sheets. Starting and ending residues from tested constructs are indicated with red arrows for insoluble, yellow for low solubility and green arrows for soluble expression.

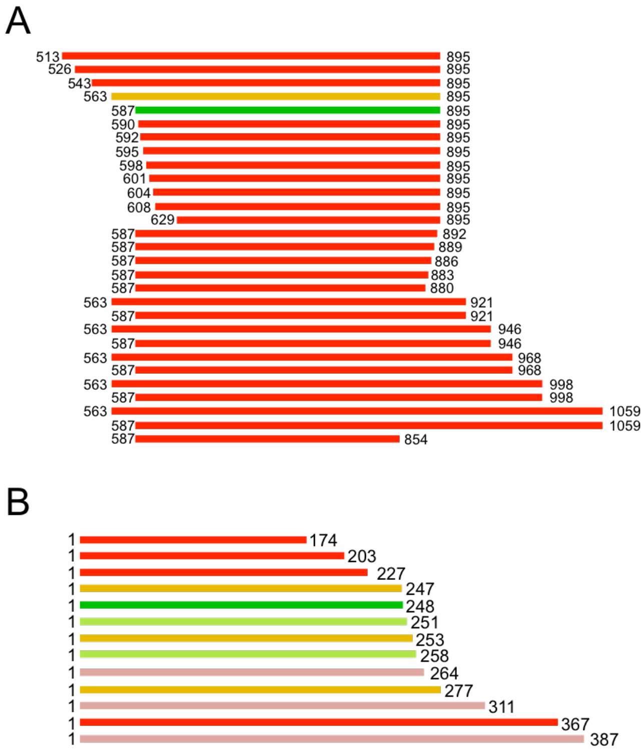

Figure S2. Constructs tested for soluble expression in *Escherichia coli* for (A) the CCHFV endonuclease and (B) the RVFV endonuclease. Residue ranges are indicated, colored according to solubility (red: insoluble expression, light red - yellow - light green: low solubility with increasing yields, green: soluble expression).

```
>RVFV   MDSILSKQLVDKT.GFVRVPIKHFDCTMLTLALPTFDVSKMVDRITIDFNLDDIQGASEI  59
>SFTS   MNLEVLCGRINVENGLSLGEPGLYDQIYDRPGLPDLDVTVDATGVTVDIGAVPDSASQLG  60   Phenuiviridae
>UUKV   MLLAICSRTNRQQ.GLNCPPAVTFTSSHMRPPIPSFLLWTEGSDVLMDFDLDTIPAGSVT  59
>PTV    MESLLRKQTINNE.GFFKPELRHSDHDLLNADLPTFLVERSSSKIIIDLNLDSLNPNSTV  59
>ANDV   MEKYREIHQRVRDLAPGTV.........................................  19   Hantaviridae
>SEOV   MEKYREIHRDLKEFTINSL.........................................  19
>LACV   MDYQEYQQFLARINTARD..........................................  18   Peribunyaviridae
>MAGV   MEDQIYDQYIKRIQSAKT..........................................  18

                            ▼
>RVFV   GSTLLPSMSIDVEDMANFVHDFTFGHLADKT....................DRLLMREFP  99
>SFTS   SSINAGLITIQLSEAYKINHDFTFSGLSKTT....................DRRLSEVFP 100   Phenuiviridae
>UUKV   GSSIGPKFKIKTQAASSFVHDFTFAHWCDAS....................DMPLRDHFP  99
>PTV    GSSLTSVIEIPEKNLMNMVHDITFGHIADST....................DVKLSSKFG  99
>ANDV   ...SALECIDLLDRLYAVRHDLVDQMIKHDWSDNKDVERPIGQVLLMAGIPNDIIQGMEK  76   Hantaviridae
>SEOV   ...TAVECMDYLDRLYAVRHDIVDQMIKHEWSDNKDSEEPISKVLLFAGIPNNVITALEK  76
>LACV   ....ACVAKDIDVDLLMARHDYFGRELCKSL..........NIEYRNDVPFIDIILDIRP  64   Peribunyaviridae
>MAGV   ....ATVAKDISTDILEARHDYFGRELCSAI..........GIEYKNNVLLDEIILDVVP  64

                             ▼            ▼
>RVFV   .........MMNDGFDHLSPDMIIKTTSGMYNIVEFTTFRGDERGAFQAAMTKLAKYEVP 150
>SFTS   .........ITHDGSDGMTPDVIHTRLDGTIVVVEFSTTRSHNIGGLEAAYRTKIEKYRD 151
>UUKV   .........LVNDTFDHWTPDFISQRLDGSKVVVEFTTNRSDQEQSLISAFNTKVGKYEV 150   Phenuiviridae
>PTV    .........IVGDGYDHLSPDMIVETTSGSYIVVEFTTFRGSERGCLNAAKDKFAKYQIA 150
>ANDV   KIIPNSPSGQVLKSFFRMTPDNYKITG.NLIEFIEVTVTADVSRGIREKKIKYEGGLQFV 135   Hantaviridae
>SEOV   KVIPDHPSGKTLRSFFKMTPDNYRITG.SLIEFVEVTVTADVDKGIREKKMKYELGLRYL 135
>LACV   ......EVDPLTIDAPHITPDNYLYIN.NVLYIIDYKVSVSNESSVITYDKYYGLTRDIS 117   Peribunyaviridae
>MAGV   ......GVNLMNYNIPNVTPDNYIWDG.DFLIVLDYKVSVGHDSTEITYKKYTSLILPVM 117

>RVFV   CENRSQ....GRTVVLYVVSAYRHGVWSNLELEDSEAEEMVYRYRLALSVMDELRTLFP. 205
>SFTS   PISRRVDIMENPRVFFGVIVVSSGGVLSNMPLTQDEAEELMYRFCIANEIYTKARSMDAD 211
>UUKV   ALHNRSTT...SSILFGVVVVSETTVVTNLNLNQQEVDELCFRFLVARAVHLEMTTKMII 207   Phenuiviridae
>PTV    CENRSRT....APVSLYVIAVHRDGLWTNMTMSQDEVNELVFRYRMALSIHEESRKICPE 206
>ANDV   EHLLETESRKGNIPQPYKITFSVVAVKTDGSNISTQWPSRRNDGVIQHMRLVQADINYVR 195   Hantaviridae
>SEOV   EQELMTFFHRGELQNPYKITFKVVAVRTDGSNISTQWPSIRNDGVVQYMRLVQAEISYVR 195
>LACV   DRLS.....IPIEIVIVRIDPVSKDLHINSDRFKELYPTIVVDINFNQFFDLKQLLYEKF 172   Peribunyaviridae
>MAGV   EELG.....IPSEIAIIRANPVTYQISIIGENFKARYPNIPIQLDFSKFFELRKMLLDKF 172

>RVFV   .ELSSTDEELGKTERELLAMVSSIQINWSVTESVFPPFSREMFD   248
>SFTS   IELQ.KSEEELEAISRALSFFSLFEPNIERVEGTFPNSEIKMLEQFL 257   Phenuiviridae
>UUKV   PEYD..DEDEDKRSREVKAAFHSVQPDWNVTEANFAPFSRRMFSNFA 254
>PTV    LTDD..DSDLSKAERELLGTLSFIDLDWNKTEKIFPMFKKAVFDNF  250
>ANDV   EHLI                                            199   Hantaviridae
>SEOV   EHLV                                            199
>LACV   GDDEEFLLK                                       181   Peribunyaviridae
>MAGV   ADDEEFLLM                                       181
```

Figure S3. Secondary structure based sequence alignment of endonuclease sequences from viruses of the *Phenui-*, *Hanta-* and *Peribunyaviridae* families. α-helices are shown with red letters, β-sheets with blue letters. Triangles mark conserved active site residues. All depicted sequences can be predicted to be a His+ endonuclease, according to the existence of a conserved histidine in a large α- helical region. (RVFV - Rift valley fever virus; SFTS - Severe fever with thrombocytopenia syndrome virus; UUKV - Uukuniemi virus; PTV - Punta toro virus; ANDV - Andes virus; SEOV - Seoul virus; LACV - La Crosse virus; MAGV - Maguari virus)

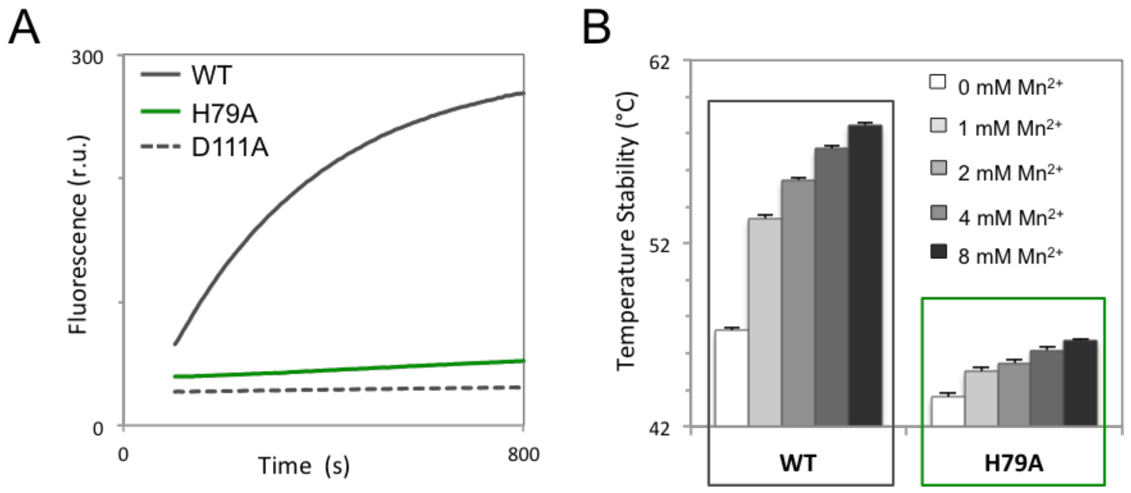

Figure S4. The RVFV endonuclease belongs to the class of His+ endonucleases. Purified RVFV endonuclease WT, D111A and H79A mutant tested for nuclease activity and manganese binding. **(A)** The fluorescence increase upon degradation of 0.1 µM 12mer FRET substrate (6-FAM-5'AAAAAAAAAAAA3'-BHQ-1) by 1.0 µM wild type protein in presence of 2 mM $MnCl_2$ was measured compared to H79A and D111A mutant. **(B)** The thermal stabilization of 3.5 µM RVFV endonuclease wild type and H79A mutant protein by $MnCl_2$ in increasing concentrations was measured by thermal shift assay. Columns represent the temperature at half of the maximum fluorescence intensity obtained for each condition. Shown is the mean of three independent measurements +/- standard deviation.

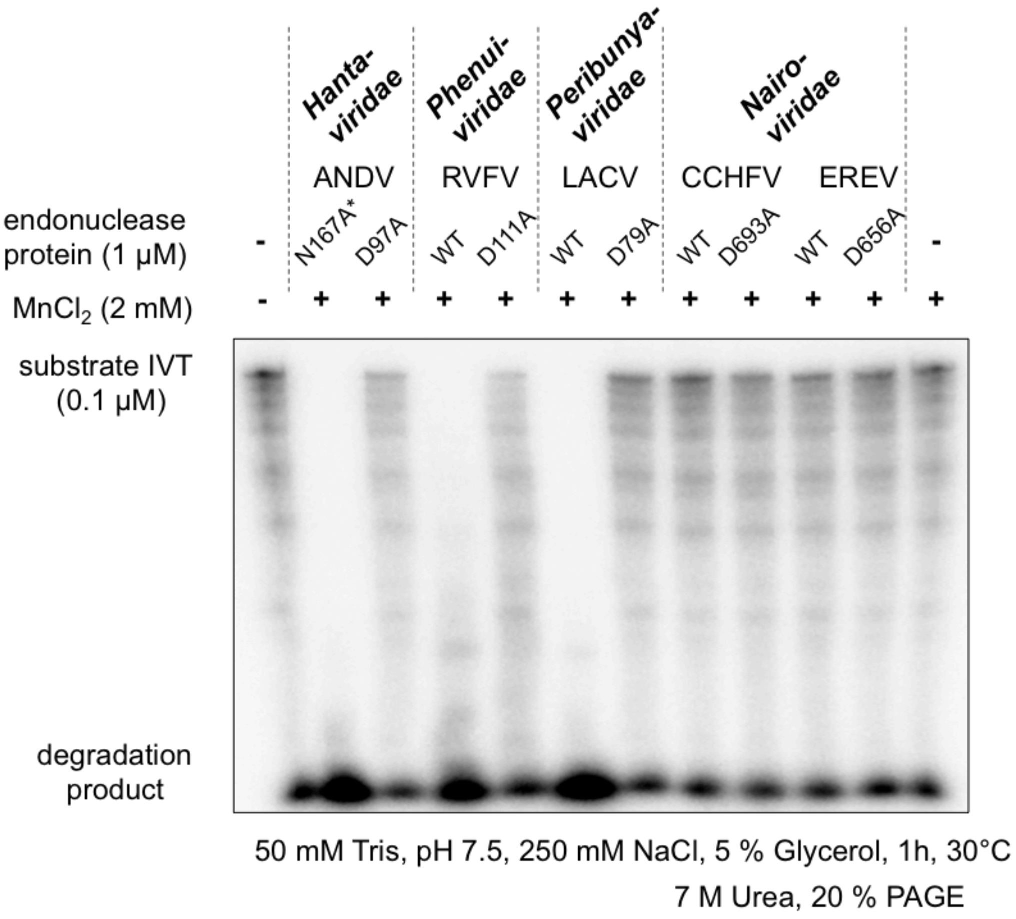

Figure S5. Comparison of IVT substrate degradation by 5 different endonuclease proteins of the order *Bunyavirales*. 20 nM of purified wildtype and active site mutants of each endonuclease protein were incubated with 2 nmol of *in vitro* transcribed RNA substrate in the presence of 2 mM $MnCl_2$ for 1 h at 30°C. Reaction products were separated by denaturing PAGE and visualized by autoradiography.
* expressable mutant, equivalent to wild-type (1).

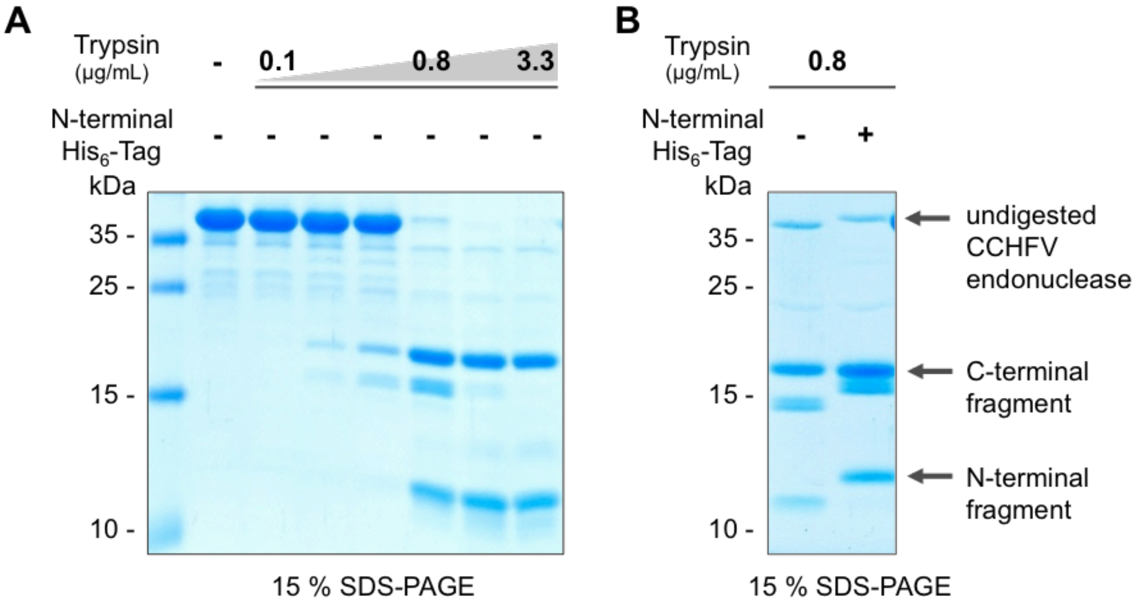

Figure S6. Limited Proteolysis of CCHFV endonuclease protein and fragment characterization. (A) CCHFV endonuclease protein without His-tag at a concentration of 0.1 mg/mL was subjected to limited proteolysis with trypsin for 1 h at 4°C. The Coomassie-stained SDS gel shows the resulting fragmentation pattern with trypsin concentrations from 0.1 µg/mL to 3.3 µg/mL. Three fragments are visible at 0.8 µg/mL trypsin, the middle one is not visible at 1.6 µg/mL trypsin. **(B)** CCHFV endonuclease with or without N-terminal His-tag was subjected to 0.8 µg/mL trypsin proteolysis for 1 h at 20°C. The gel shows the fragmentation pattern of both. The two lower bands of the His-tagged protein have a higher molecular weight indicating the His-tag to be present. The Coomassie-stained bands are assigned according to mass spectrometry analysis.

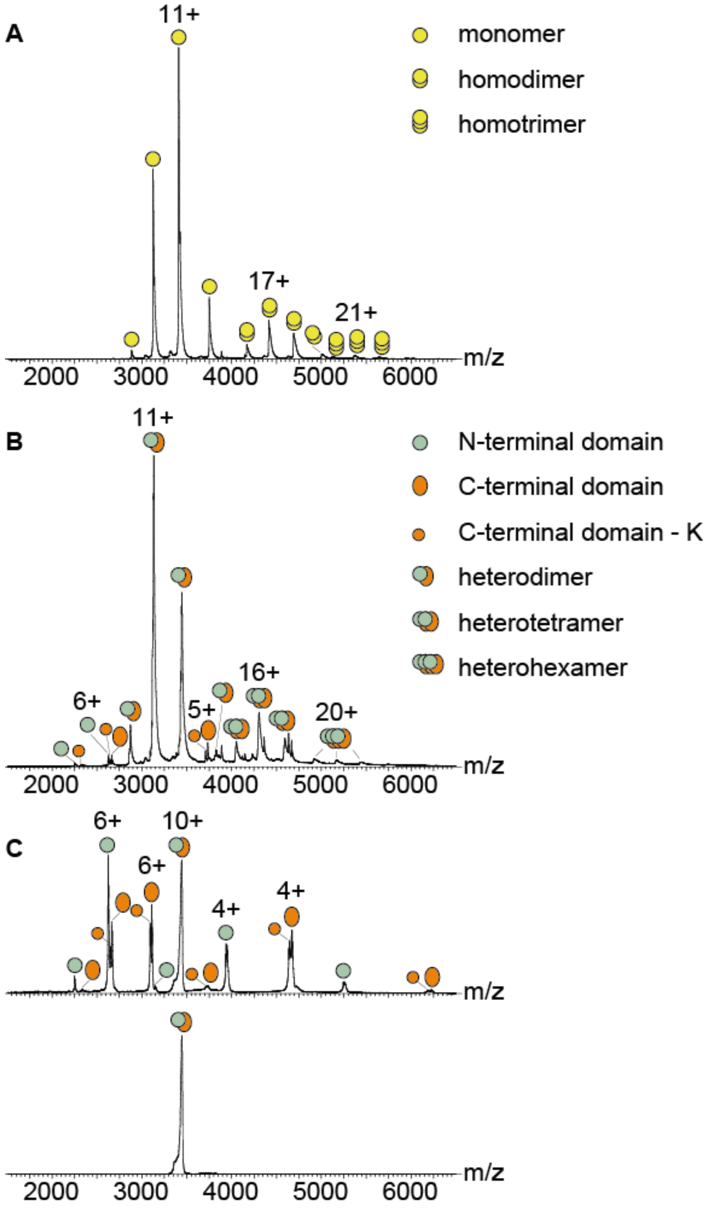

D

	Species	Theor. mass	Exp. mass	St. dev.	FWHM
undigested CCHFV endo	monomer	37,498	37,498	5	155
	homodimer	74,996	75,060	50	500
	homotrimer	112,494	112,580	70	1,020
trypsin digested CCHFV endo	N-terminal domain	15,730	15,729	1	29
	C-terminal domain	18,668	18,667	2	36
	C-terminal domain –K	18,540	18,539	1	78
	heterodimer	34,398	34,430	50	280
	heterotetramer	68,796	68,890	20	580
	heterohexamer	103,194	103,370	40	1,980

Figure S7: **Trypsin digestion of the CCHFV endonuclease domain monitored by native MS.** ESI-MS of 10 µM undigested (**A**) and digested (**B**) CCHFV endonuclease domain in 250 mM ammonium acetate, pH 7.4 recorded at 10 V acceleration into the collision cell. Monomeric endonuclease domain was identified as most abundant species in case of the undigested sample whereas a heterodimeric complex of C-terminal and N-terminal subunit was detected in the trypsin-treated sample. (**C**) MS/MS analysis of 10^{+}-charged peak at 3375 *m/z* performed on 50 µM digested endonuclease domain at 50 V and 100 V for collision determines accurate masses of the two subunits by which means the boundaries of the excised

fragment were derived (cleavage after R704 and R733). Simultaneously, this experiment shows the presence of a shorter version (-K, lacking K734) of the C-terminal domain resulting from cleavage at an alternative site next to R733. Thus, two different C-terminal domains result from cleavage at two adjacent sites, R733 and K734, respectively. (**D**) Masses of different protein species from undigested and trypsin-digested CCHFV endonuclease domain were determined from at least three independent MS measurements. They are listed together with the respective values for standard deviation and average full width of the peak at half maximum (FWHM) along with the theoretically calculated molecular weight. FWHM values are given for the whole peak area where individual species were not fully resolved. All listed values are in Da.

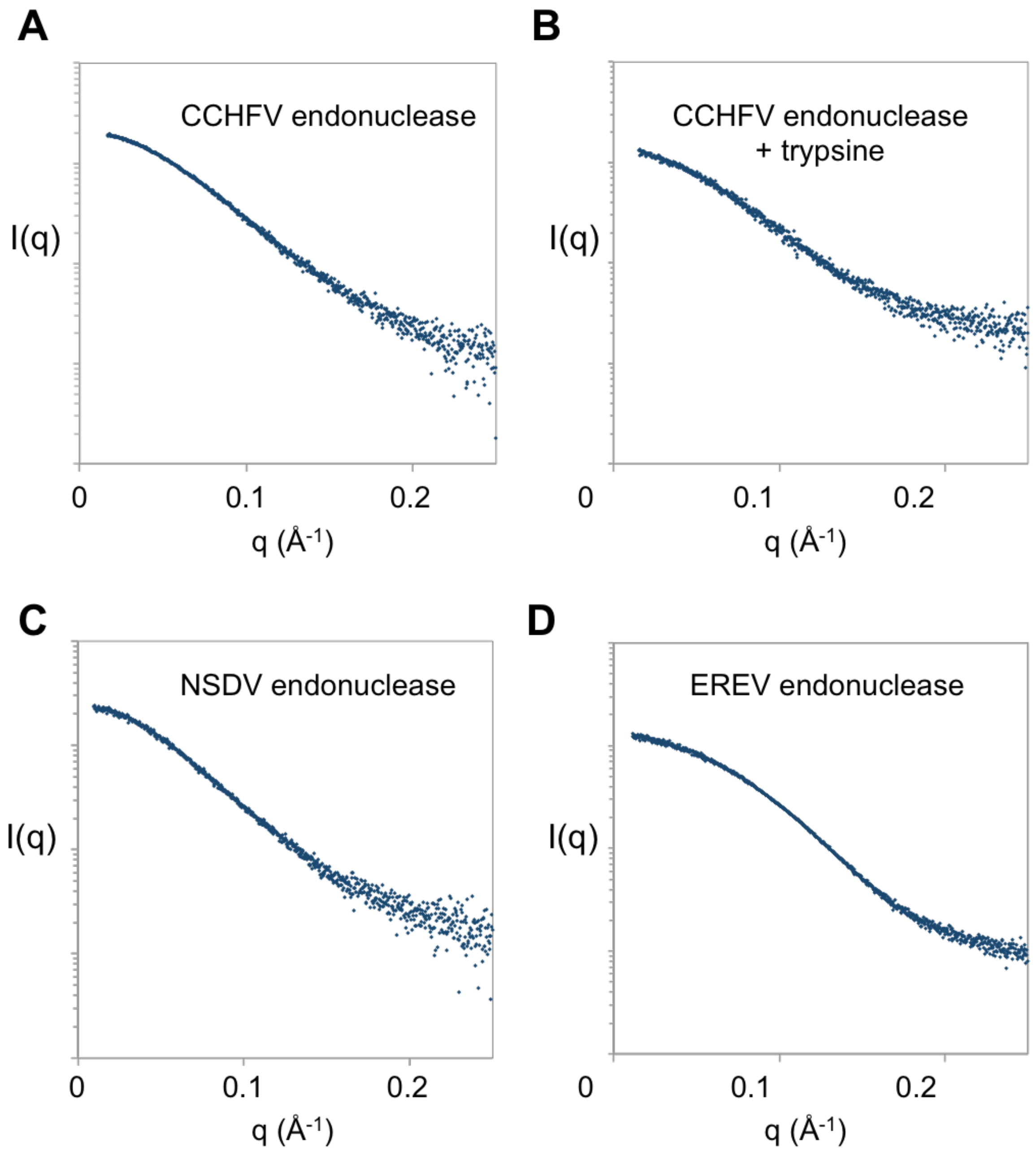

Figure S8. SAXS scattering curves of nairovirus endonuclease proteins. The plots of experimental scattering data were obtained by measuring 3 different protein concentrations and are represented for CCHFV endonuclease before trypsin digestion in **(A)** and after digestion with trypsin **(B)**. The curves for NSDV endonuclease and EREV endonuclease are represented in **(C)** and **(D),** respectively.

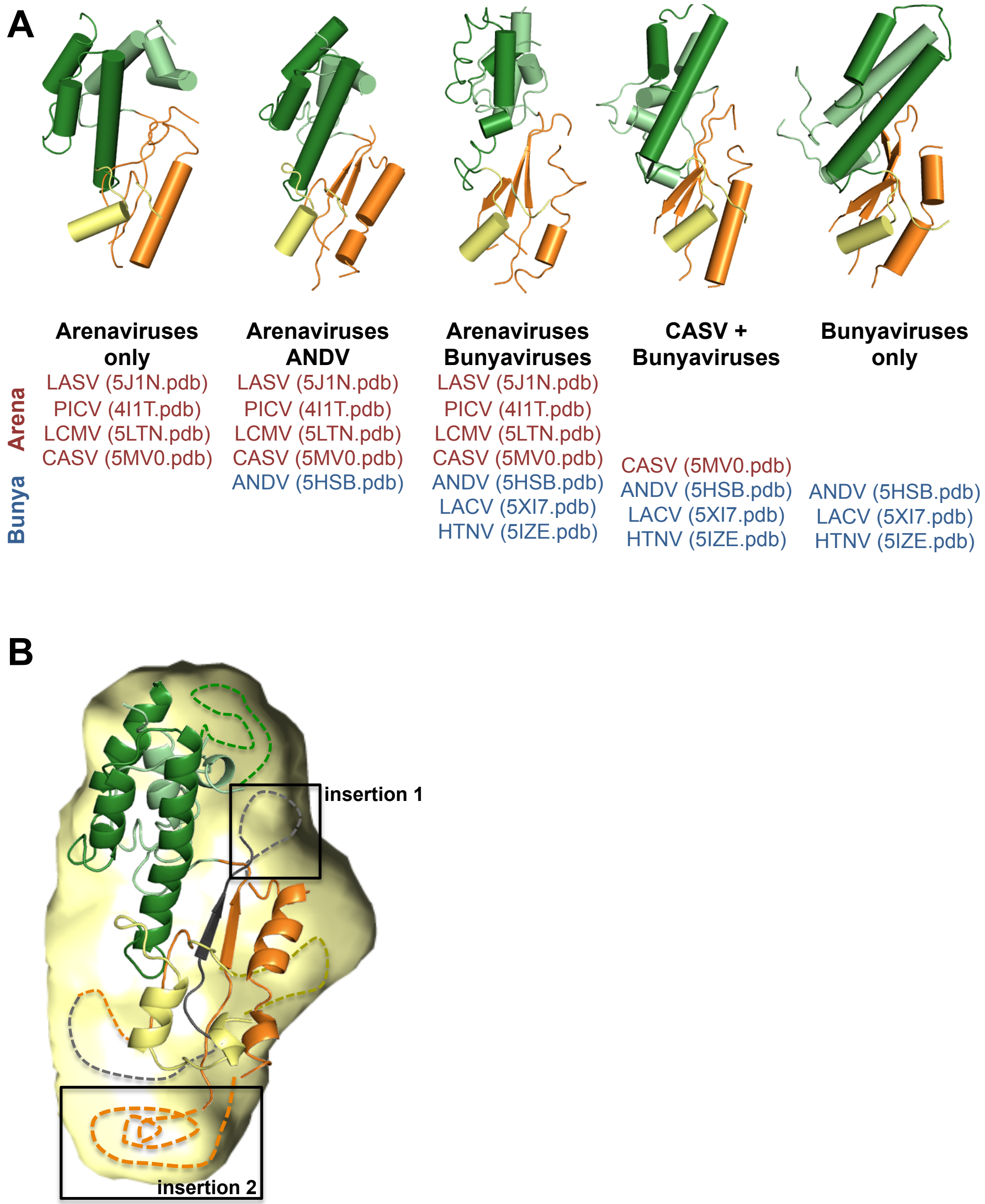

Figure S9. Homology-based structural models of the core of the CCHFV putative endonuclease structure and comparison to the SAXS envelope. (A) Different computational models of the CCHFV putative endonuclease domain created by MODELLER using varying input parameters show the high variability in the resulting models. The atomic structures used as templates for the different models are given, including the corresponding pdb-codes. Regions, which were extremely variable between the models and did not contain any secondary structure elements are not shown. **(B)** The core of the CCHFV putative endonuclease structure, modeled using a set of atomic structures of the endonuclease from Arenaviruses and the Hantavirus Andes virus, is shown superimposed with the CCHFV putative endonuclease SAXS envelope (yellow). Regions, which are not shown in (A), are sketched with dashed lines. The regions with non-predictable secondary and tertiary structure in the homology model (dashed lines) nicely superpose with areas of additional electron density in the SAXS envelope. The peptide cleaved out by trypsin in the limited proteolysis experiments is shown in grey. Insertions 1 and 2 as mentioned in the text are marked. (LASV - Lassa virus, PICV - Pichinde virus, LCMV - lymphocytic choriomeningitis virus, CASV - California Academy of Science virus; ANDV - Andes virus; HTNV - Hantaan virus; LACV - La Crosse virus)

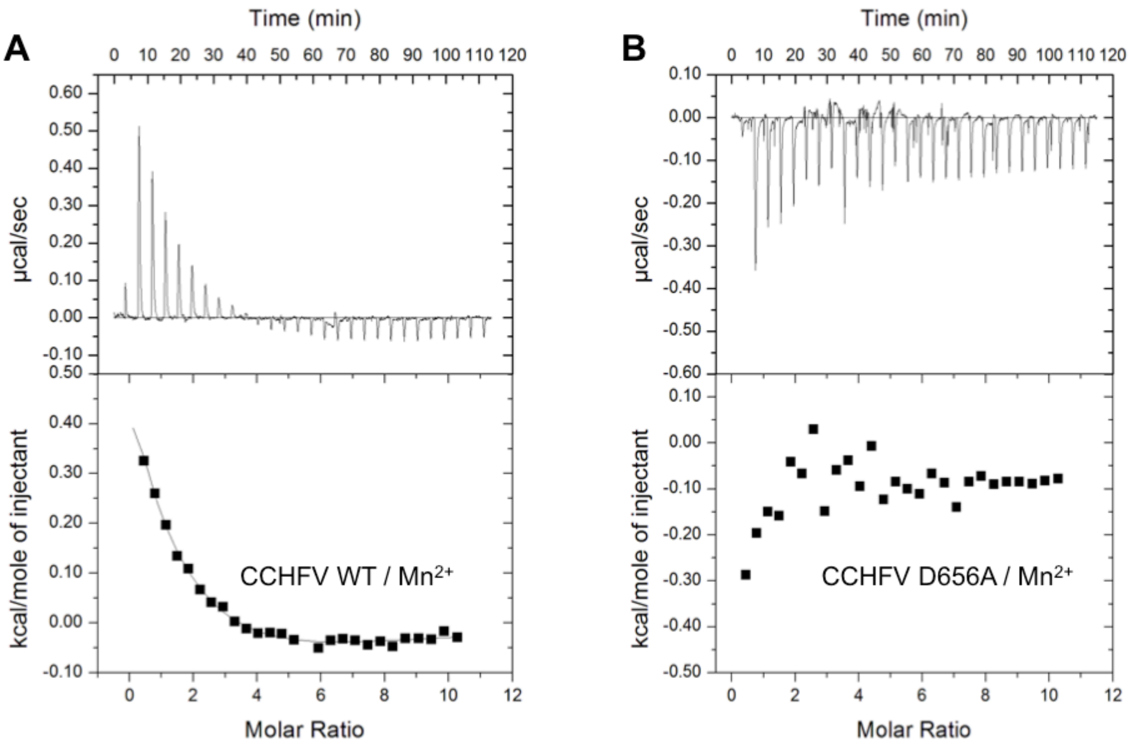

Figure S10. Affinity to manganese ions is strongly reduced in the CCHFV E656A mutant compared to the wild type. 100 µM of wild type **(A)** or D656A mutant **(B)** endonuclease protein was titrated with 12.5 µL injections of 8 mM $MnCl_2$ in a buffer containing 50 mM Tris, pH 7.5, 150 mM NaCl and 2.5% glycerol at 20°C using a VP-ITC calorimeter. The upper plot shows the binding isotherm and the lower plot shows the integrated values of each peak.

Supplemental Reference

1. Fernandez-Garcia, Y., Reguera, J., Busch, C., Witte, G., Sanchez-Ramos, O., Betzel, C., Cusack, S., Gunther, S., and Reindl, S. (2016) Atomic Structure and Biochemical Characterization of an RNA Endonuclease in the N Terminus of Andes Virus L Protein. *PLoS Pathog* **12**, e1005635
